# Renegotiating autonomy and safety: disentangling tensions emerging for older adults living with smart monitoring systems

**DOI:** 10.1038/s44401-026-00154-4

**Published:** 2026-10-08

**Authors:** Laura Detels, Mingsong Zhai, Zhao Cai, Manuel Trenz

**Affiliations:** 1 University of Goettingen, Goettingen, Germany; 2 University of Nottingham Ningbo China, Ningbo, China

**Keywords:** Health care, Psychology, Psychology

## Abstract

Smart monitoring systems (SMS) are increasingly deployed in community-based care to empower older adults to live safely and independently. However, existing research largely treats monitoring as a process in which continuous data collection and real-time alerts are intended to translate into improved outcomes. What remains underexplored is how older adults experience monitoring and how it shapes their behavior over time. In this qualitative longitudinal study, 21 older adults are accompanied from SMS installation for more than one year, with individual semi-structured interviews conducted across four data collection phases. Findings reveal that older adults actively anticipate how the system interprets their behavior, producing three responses: reactance, that is, disengaging from devices to reclaim autonomy over safety; preemptive behavior, for instance, restructuring daily routines to avoid triggering alerts constraining their autonomy and safety; and performative compliance, that is, managing behavior to satisfy caregivers rather than personal needs, leading to a loss of autonomy. These constitute autonomy-safety value tensions: a technology designed to advance both values instead produces conditions under which protecting one systematically undermines the other. To this end, this study provides empirical insights into unintended consequences and a conceptual model explaining how value tensions emerge, with implications for practitioners and caregivers.

## Introduction

Global populations are aging at a rapid pace, putting healthcare systems under pressure to provide dignified elderly care^1,2^. Institutionalized care for older adults is costly, labor-intensive, and opposed to the need for autonomous and safe aging in place, that is, maintaining independence within the familiar surroundings of one’s own home^3^. Consequently, policy makers, care providers, and technology developers have sought alternatives that follow the dual objective of empowering individual autonomy and physical safety in later life^4,5^.

**Fig. 1:**
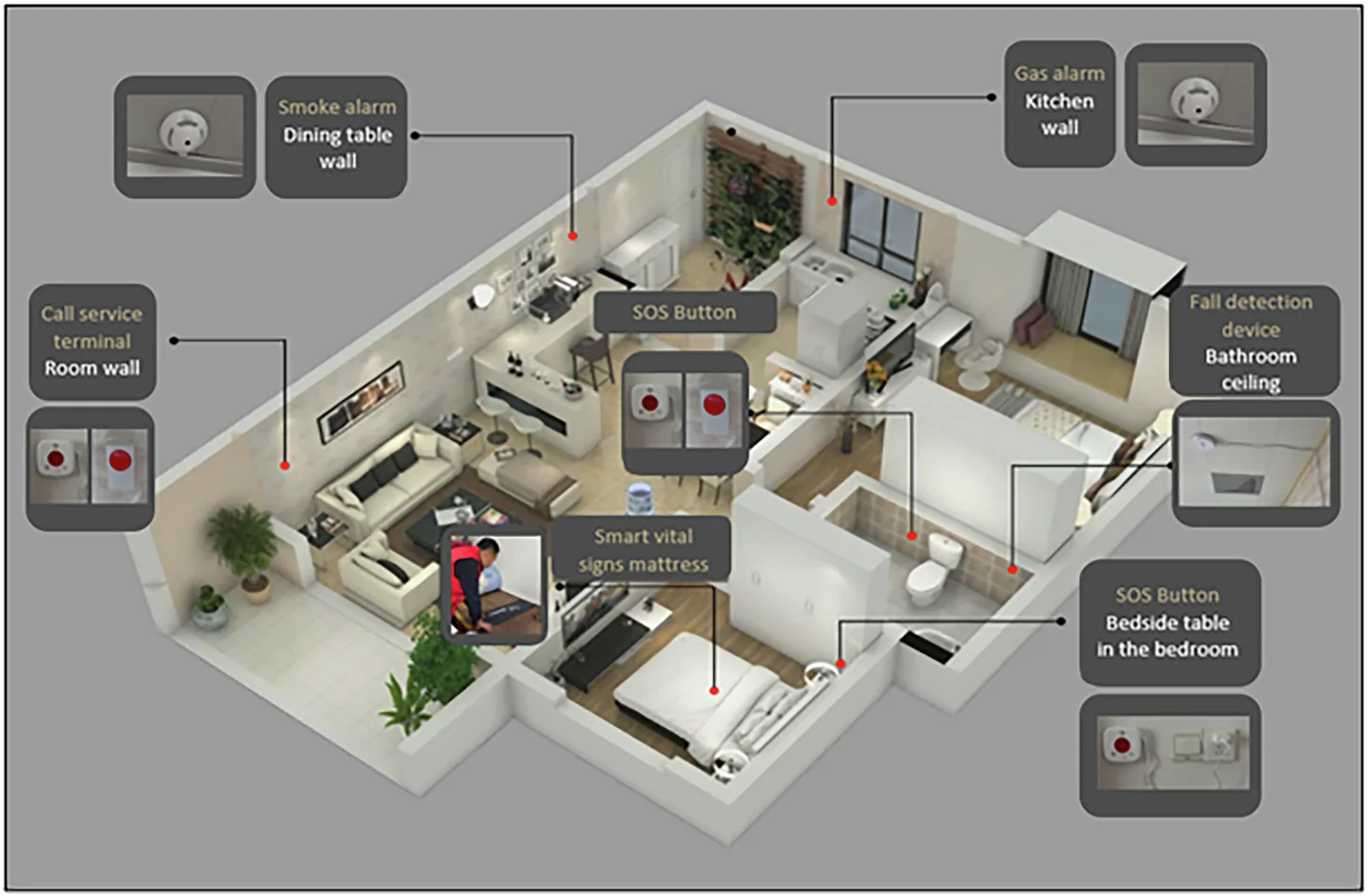
Installed smart monitoring system under investigation.

The decline in cost for digital healthcare technologies has given rise to a promising solution: smart monitoring systems (SMS)^6,7^. These systems leverage environmental sensor networks, including fall detection and movement sensors, smoke detectors, emergency buttons, and smart mattresses (see Fig. 1), to unobtrusively, yet continuously, monitor activities and vital signs of their users^8^. By transmitting collected data to respective responders, such as professional or informal caregivers, SMS enable early detection of critical events and timely intervention^9,10^. Hence, these systems are increasingly installed in living environments explicitly aiming to empower older adults, for example, by ensuring that medical monitoring is provided not only in the presence of a caregiver but also during the largest parts of the time the older adult spends on their own^11–13^.

Yet, this objective becomes fragile once SMS are understood not as purely technological infrastructure but as sociotechnical arrangements that actively reconfigure individual agency^7,14^. For instance, fall detection sensors installed across rooms of an apartment do not merely register emergencies. They also render movement patterns continuously visible to relatives and caregivers, transforming private activity into monitored events^15^. This tension has been anticipated in design discourse. For instance, the speculative design project *Uninvited Guests*^16^ portrays an older adult quietly circumventing the connected devices their children have installed to look after them, capturing how well-intentioned technologies can become adversaries in the very home they are meant to secure. Living with SMS therefore reconfigures not only how individuals perceive their domestic environment but also how they position themselves in relation to it.

Still, prior health information systems (IS) research has primarily focused on enhancing technological performance to improve care delivery^17–19^. This approach assumes that users experience monitoring in a passive process, where data flows unobtrusively to caregivers and alerts trigger responses, enabling older adults to continue living safely and independently^5,20,21^. What remains underexplored is how older adults actually experience living under continuous monitoring in their own homes and how it shapes their feelings, their daily routines, and the choices they make. While existing studies tend to assume that living with monitoring technology intended to empower their users leaves residents’ behavior largely unchanged, the lived experience of being continuously observed has yet to be examined in depth^22^.

Being designed as always-on networks embedded in domestic environments^19^, SMS risk triggering value tensions that are not merely designed into the technology but enacted through behavioral and psychological responses to it^4,23^. It is this very dynamic between continuous monitoring and the everyday choices of those living with the SMS that puts the understanding of joint empowerment of safety and autonomy under pressure and calls for careful empirical and theoretical reconsideration. Hence, we ask, *what are unintended consequences that emerge when older adults live with smart monitoring systems in community-based environments, and how do they produce autonomy-safety value tensions?*

In this study, we address the above research question by investigating the lived experiences of older adults using SMS over an extended period of time. Drawing on a qualitative longitudinal research (QLR) design^24–27^, we accompany 21 older adults in community-based housing from the point of system installation through more than one year, interviewing 19 of them across four subsequent phases between December 2024 and March 2026. Using elements from grounded theory methodology^28,29^ and value tensions as a sensitizing lens, we analyze how smart monitoring systems shape everyday decision-making and routines over time^30,31^.

Our findings reveal that living with SMS is far from passive. Across the data collection and analysis, older adults mention awareness of being observed and anticipate how their behavior could be interpreted by the system and its responders. This awareness leads to three distinct unintended consequences: reactance, in which older adults disengage from using the SMS as a whole or several devices; preemptive behavior, in which residents modify routines to avoid triggering alerts; and performative compliance, in which users maintain engagement producing data that is anticipated to satisfy their caregivers. Together, these responses produce autonomy-safety value tensions. We find that a technology designed to secure both values simultaneously also leads to trade-offs or the constraint of both values.

This study contributes to the academic and practical discussion on SMS for elderly care in three ways: two contributions to research and one to practice. First, addressing the lack of longitudinal research on SMS use, we provide an in-depth empirical account of living with SMS, capturing the behavioral and psychological mechanisms through which unintended consequences unfold for older adults over time. Second, we offer a conceptual contribution by identifying psychological and behavioral adjustment as mechanisms that produce autonomy-safety tensions, thereby challenging the prevailing assumption that SMS can jointly optimize autonomy and safety as an inherently empowering outcome. Third, for practice, we derive implications for designing monitoring systems that support communication, agency, and negotiated control for older adult users.

To examine how SMS shape the everyday lives of older adults, we draw on literature from two interconnected perspectives^25,32^, namely the sociotechnical understanding of smart monitoring systems^14,33^ and the concept of value tensions^34,35^. Rather than treating them as purely technological devices for delivering care, we approach SMS as sociotechnical arrangements that actively structure the social lives of older adults. Together, these perspectives provide us with theoretical lenses and allow us to investigate older adults’ decision-making and underlying psychological mechanisms^25,31^.

Smart monitoring systems in elderly care are intended to empower older adults to live independently and safely^12,13^. In this regard, empowerment refers to conditions and processes that strengthen the individual’s sense of self-determination and self-efficacy while reducing feelings of powerlessness^36–38^. In the context of elderly care, SMS are expected to provide safe living conditions or timely intervention that in turn ensures a sense of independence and control^12^. Understanding smart monitoring systems as empowering devices provides us with vocabulary for describing what these systems are intended to achieve. This understanding allows us to ask whether the unintended consequences of living with SMS remain local frictions or accumulate into disempowerment, that is, a process in which individuals lose control over decisions, information, or actions concerning their own lives^39^, running counter to the technology’s intent.

Moreover, monitoring systems are understood to be tools that collect and transmit data^40,41^. However, technology and human action are not separate but mutually constitutive, as the technology’s inherent functions shape how users act^7,14^. Users’ actions in turn give meaning to and reshape what the technology does in practice^23,42^. Following this understanding, SMS features, specifically the data they collect, when they trigger alerts, and to whom they provide data, actively shape older adults’ lived experiences^33^. Thus, SMS reconfigure the relationship between the individual and their living environment^43^. Therefore, this perspective offers us a lens to uncover the technology’s intended design and investigate psychological and behavioral outcomes that emerge through its everyday use^25,31^.

The concept of value tensions provides us with vocabulary for understanding and conceptualizing unintended consequences that emerge when older adults live with SMS. Value tensions arise when goals that appear complementary turn out to conflict when pursued simultaneously^44,45^. This is specifically the case when a technology’s design or its use brings competing values into conflict, forcing trade-offs that are not intended^4^. Crucially, value tensions do not require a technology malfunction but rather that its intended functioning generates contradictory outcomes or byproducts, therefore, uncovering moments and mechanisms through which one value is advanced at the expense of another.

In healthcare contexts, this simultaneous pursuit of values produces contradictions structurally embedded in care delivery^45,46^. These contradictions emerge when designed intentions collide with lived practice^45^. In elderly care specifically, such tensions are amplified by relationships of dependency and vulnerability^34^. Thus, health systems used in elderly care are prone to producing value conflicts not only between stakeholder groups but also within individuals simultaneously^34,47^. An older adult, for example, recognizing that location tracking protects their safety if they fall outside, can simultaneously experience this very same technology as an infringement on their privacy^47^. These contradictions are inherent in the technology’s function of continuous monitoring, that is, providing continuous data about one person to another^4,45^. Overall, literature on value tensions allows us to conceptualize mechanisms through which one value is advanced at the expense of another.

Taken together, these perspectives allow us to understand the sociotechnical functions and value conflicts under which unintended consequences can emerge when older adults live with SMS. What both perspectives leave underspecified, however, is the older adult’s own agency in this process: how, over time, older adults actively anticipate, interpret, and act upon the very mechanisms that render them visible to caregivers and relatives. Existing accounts tend to frame such responses as barriers to acceptance or byproducts of vulnerability, rather than as purposeful strategies of managing one’s own visibility. A growing body of critical Human-Computer Interaction (HCI) and aging scholarship argues for precisely this shift in perspective, positioning older adults as agentic actors who manage their everyday sociotechnical environments^48^. Within this stream, disengagement has been reframed as a deliberate deployment of distrust rather than as failed acceptance^49^, and the small material rearrangements older adults make in their homes have been shown to be autonomy-enabling rather than deviant^50^. What remains open is how such strategies unfold over time in arrangements where the technology continuously transmits data to third parties. Addressing this gap, we examine how older adults actively manage the conditions under which they are monitored, and how this management itself produces and reshapes autonomy-safety tensions over time.

## Results

We report our findings in the order in which they emerge across the four phases of our fieldwork, from the installation of the SMS through more than one year of living with it. The methodological approach underlying these phases is detailed at the end of this article. Analyzing our data, we find that living with SMS fundamentally shapes older adults’ everyday lives. Accompanying our informants provides us with insights on how they encounter, live with, and adapt to the presence of continuous monitoring in their homes. While the following accounts revolve around concrete material details, namely the smart mattress, the alarms, and the placement of devices, we understand these not as findings in themselves, but as traces of the deeper behavioral and psychological mechanisms through which older adults negotiate autonomy and safety under continuous monitoring. Therefore, our analysis reveals that monitoring is far from passive. The system’s presence is felt and interpreted by its users even when no alert is triggered or no intervention occurs. Participants are aware of and actively perceive the possibility that relatives or caregivers may intervene. This realization provokes older adults to adjust routines in response to how they believe the system interprets their behavior.

### Phase 1: Installation and demonstration—acceptance grounded in institutional trust and safety concerns

In phase 1, we capture our informants’ initial encounters with the SMS at the moment of installation. Across informal conversations, acceptance is the dominant orientation, shaped primarily by two interrelated factors: trust in the institution organizing the deployment and personal health concerns tied to the experience of living on one’s own.

When installing the SMS, participants consistently express willingness to use the system and they report confidence in its potential to support safe and independent living. This initial acceptance is grounded in institutional trust or as P4 puts it, *“Anyway, we trust it, we believe it’s trustworthy”* (P4, phase 1). Moreover, reliability plays an important role when the systems are installed, as *“these people are reliable, not some random messy people out in society”* (P13, phase 1). Furthermore, a pragmatic orientation toward aging and the recognition that the technology is offered free of charge through a trusted community organization is central for the older adults, as P3 puts it, *“The community cares about us, right? That’s a good thing”* (P3, phase 1).

Similarly, another participant with no prior experience with health technology states that trust in the organizing institution is sufficient to overcome any uncertainty about the system itself, *“I never had any concerns. I had no mental barriers about using it […] because I trust this system. Since it’s organized by the center [the community management center], we trust you. It’s not like some random thing being handed out”* (P5, phase 1).

Alongside institutional trust, participants mention personal health concerns and lacking safety when living alone. Several informants describe prior health events, such as falls, cardiovascular episodes, and hospitalizations as reasons they see the SMS as necessary, or as P6 explains, *“Yes, I want to use it because I have health issues. In the past three years, I have fallen five times, all in this house”* (P6, phase 1). Adding to this, P19 mentions, that they *“already have a heart condition, and this system provides extra safety”* (P19, phase 1). Moreover, participants also describe the system’s value in explicitly relational terms. Not only protecting themselves, but also reducing the burden on adult children who live far away. One participant explains their motivation to use the SMS as follows, *“If something happens while I’m in bed, the alarm system will work. Even when going out, there’s still protection. If someone falls, even if they lose consciousness, this safety system will be in place”* (P16, phase 1). Being asked about the interaction with the caregiver, for instance their children, they elaborate, that *“they used to tell me not to lock the door from the inside because if something happened, they wouldn’t be able to get in. But now, with this system, they don’t have to worry. Even if they’re not here, they can still receive updates”* (P16, phase 1). We find participants recognizing the SMS’s intent to enhance safety and enable their users’ independent living.

Despite the broadly positive reception, phase 1 conversations, however, also reveal early signs of complexity. Informants who voice appreciation for the system are not entirely uncritical. Several informants express hope that the SMS would simply work as promised but remain pragmatic about its limitations. One participant captures a widely shared conditional trust, mentioning that *“I do trust this system. However, as technology develops, there will always be some imperfections. These issues can only be identified and improved through actual use. But I don’t have excessively high expectations”* (P1, phase 1).

### Phase 2: Connectivity and calibration check—disengagement and reactance emerge

Eight months after installation, we uncover divergences emerging between the enthusiasm expressed at installation and how participants actually use the SMS. Patterns of selective disengagement, in particular with the smart mattress, are widespread. Our informants point out two main reasons for reducing their engagement, that are physical discomfort and alarms or even false alarms that they experience.

One reason for discontinuing the use of one device of the SMS, that is, the mattress, is stated to be its discomfort. P7 mentions that *“it’s often pressed underneath me. In summer, I can’t use those thick blankets on the bed. When I lie there, it’s right underneath with almost no buffer. In summer we use bamboo mats; cool bamboo, right? It’s not like cotton. In that situation, once the weather got warm, I could only turn it off”* (P7, phase 2). Specifically, the device’s rigid fiber-optic material is felt through bed coverings, and its placement directly underneath users’ bodies disrupts sleep or as P1 puts it, *“it’s useless. […] Sometimes in summer it feels uncomfortable; in winter I’m lazy; sometimes I’m too lazy to lay it down again”* (P1, phase 2). Our informants report not only that they occasionally unplug the technology, but also that they simply do not use it again, stating *“I used it before, but then I unplugged it. It was giving off heat”* (P4, phase 2).

In addition to physical discomfort, however, participants state another, technology inherent reason why they decide to disengage with several devices of the SMS. Specifically, because the mattress relies on pressure-based detection of breathing and heart rate movement, body movement during sleep is frequently misinterpreted as an emergency event, triggering alerts. Because these false alerts disrupt family members in particular, participants disengage from the devices that produce them. *“I was asleep, and once I heard the ring, I knew what it was. They said there must have been movement, right? When you move and touch it, they [the informant’s children] call. That’s how it is. After many times, I couldn’t take it anymore. It affected my sleep at night, right? So, I forgot about it and unplugged it”* (P7, phase 2). One participant similarly describes the false alarm cascade in which the technical alarm triggers a call to their adult child, which disrupts the child’s sleep, which in turn makes the participant feel they are causing harm rather than being helped, *“after I came back from the bathroom, it triggered an alarm, and the son also received a phone call. Later, I […] unplugged it”* (P8, phase 2). For the spouse living with P8, the alarm system feels frightening rather than reassuring. They mention, *“after it shouted a few times, I unplugged it. If it scares me like that, I’d rather comfort myself. Suddenly it screams, and you don’t know what it is”* (P8’s spouse). Furthermore, participants mention protective claims and laugh off false alarms, stating, *“sometimes I even laugh. I’ll be cooking something simple, a bit of steam comes up, and then it starts calling. I laugh and say, ‘Listen, it’s calling again, bringing people from Yantai to come take care of us’”* (P3, phase 2).

We find a friction point emerging around the transmission of alarms. Participants question why alerts are sent to a caregiver or a family member rather than alarming the older adult themselves so that they would not disrupt their relatives’ sleep by notifying them of their health status. *“What I think is: the alarm shouldn’t call the son. If there’s an alarm at home, as long as I can hear it, I’m fine. I can call the son myself, right? This way it doesn’t affect his rest. If I hear it, I can call 120”* (P5, phase 2). However, the SMS is designed to route all alerts to designated caregivers, either professionals or informal caregivers, such as relatives. This decision is made on the assumption that the older adult may be unable to call for help themselves. Yet, several participants challenge this assumption, arguing that routing alerts to a distant son or daughter is both slower and more disruptive than the situation requires. P10’s spouse also frames the routing problem in relational terms that go beyond operational speed. The system’s call to the son not only delays emergency response; it disrupts the son’s sleep and creates the possibility that he will feel *“annoyed”* (P10’s spouse) by his parents.

### Phase 3A and 3B: Routine maintenance visits—conditional SMS device use

One year after installation, the system settles into the background of participants’ lives. However, participants mention a self-assessed health rationale, by which they consider themselves *“too healthy”* (P4, phase 3 A) to need the system. Across phase 3A and 3B interviews, two patterns dominate: a widespread conditional logic of use and the relocation of SMS devices. We also identify an underlying psychological reason for this behavior. Participants alter the SMS to assert their autonomy, driven by the anticipation that caregivers might otherwise perceive them as incapable of living independently. In doing so, however, they place their own safety at risk.

Rather than abandoning the SMS entirely, informants state that they develop a way of conditionally using it. For instance, they employ the mattress when they feel physically unwell and leave it unplugged or set aside when they feel healthy. This behavior reflects a pragmatic approach, but it also introduces a systemic vulnerability: the device is not active in the very moments of the unexpected emergency it is designed to address. P14 mentions, *“I still haven’t used [the mattress], because right now I don’t need that mattress to measure. Right now, my daily life is doing okay, everything is normal. If I feel uncomfortable or something, then I’ll start using it”* (P14, phase 3B). Another informant mentions that, *“they [the SMS devices] are connected, but to be honest, sometimes I unplug them. I feel like my health is pretty good, and I don’t really notice much. When it’s plugged in, it’s just by the head of the bed. Usually, I feel it’s not that great to have it there all the time. If I feel a bit off or something at night, then I plug it in”* (P8, phase 3B).

Smoke and gas detection devices, which operate as passive background sensors, are more reliably kept in use, though frequently relocated due to sensitivity. Emergency call buttons are retained even by participants who have discontinued the mattress. The 4 G wristband, however, is almost universally abandoned due to inconvenience or as P4 puts it, *“Right now it’s mainly the mattress that we use most. We also had a watch, but we haven’t really worn it. Because, you know, a watch is a watch, it’s not convenient to wear”* (P4, phase 3B). From earlier phases, we find that participants aim not to trigger alarms and prefer to be informed of any deviations themselves. Underlying is a concern about being a burden to their caregivers or giving the impression that they are unable to take care of themselves, specifically to live independently.

Overall, we observe a performative use of the devices grounded in anticipated expectations. Participants acknowledge that their relatives use the devices to keep an eye on them and to protect them (P13, phase 3B). Several report that, although they are not convinced of the devices’ usefulness for themselves, they keep using them to reassure their close ones, so they can *“understand my physical condition better”* (P4, phase 3B) and *“care about me more”* (P4, phase 3B). Asked whether they would want their children to see their sleep, heart rate, and breathing data, the same participant frames the value of the data entirely in terms of what it does for the children: *“they [the children] feel more at ease, see that Dad slept well”* (P4, phase 3B). What is produced is thus oriented less toward the participant’s own health than toward the impression it creates for others. P1 pursues the same concern in the opposite direction. Rather than producing data that reassures, they restrict what their children are able to see at all. They do not want their children to have access to their respiratory or cardiovascular data, knowing that this would worry them, and they extend this to their sleep data stating *“It’s the same issue. There’s no need for them to see it. I’ve had insomnia for a long time. This has no meaning […] it doesn’t bring any benefit, and it won’t help”* (P1, phase 3B). The underlying rationale is the same as P4’s, only inverted: *“I don’t trouble the children. I don’t bother them unless I really can’t”* (P1, phase 3B). Both participants manage what their families come to know about them, one by generating a reassuring record and the other by withholding a worrying one.

Only users with severe health issues, for instance cardiac conditions, report keeping on using the mattress for their safety. P9, whose spouse has hypertension and is approaching 90 years of age, describes the mattress as a continuous form of protection that has been in use since installation, *“it felt safer, like insurance. If something happens, it can respond in time. So, several households installed it”* (P9, phase 3 A). Here, safety takes precedence over the individual’s independence. We treat this pattern as a negative case. P9 and P14 apply the same rule to opposite effect: both make use of the mattress contingent on their own assessment of health risk, but where P14 concludes that current wellbeing makes monitoring unnecessary, P9 concludes that a spouse’s condition makes it indispensable. Where perceived health risk is high, immediate, and shared with a co-resident, the autonomy-safety trade-off does not arise in the form we describe elsewhere: safety is not experienced as a constraint on independence but as its precondition. This marks a boundary of our framework rather than an exception to it, since the mechanisms we identify presuppose, that older adults do not perceive themselves as being in acute need of the protection the system provides.

Adding to our findings from phase two, we discover that our informants adjust their behavior according to how they expect the SMS to interpret it. We furthermore find that informants not only react to false alarms, for example when triggering the smoke detection device, *“It has probably happened three times. Each time it starts beeping, I take action immediately; either cover the pot or turn on the range hood. Once it’s under control, it stops beeping”* (P4, phase 3 A) but they also preemptively adjust their routines to produce preferred outcomes. *“In the end, you can manage it [and cook on lower heat]”* (P4, phase 3B) or *“You still need to adapt to it and try not to trigger it. When I cook, I need to control the amount and heat”* (P4, phase 3B). However, our informants also describe that they actively relocate devices of the SMS, for instance the smoke detection device. As the devices cause false alarms during cooking, our participants feel prompted to relocate them, *“after it was installed, nothing really happened. But that alarm device… When cooking, even a bit of steam or cooking fumes triggers it. The smoke alarm was too sensitive, so later we removed it, and then moved it elsewhere”* (P9, phase 3 A). This way, informants undermine their own safety in order to strengthen their autonomy.

### Phase 4: Final technical review—from emergency device to daily mirror

By phase 4, a material change for the SMS occurs. Community engineers install mini programs connecting the smart mattress to a smartphone interface across participating households. Information on new functions is provided as printed material and in informational conversations. With the installation, participants, for the first time, have direct access to their own nighttime health data: sleep quality scores, heart rate and breathing graphs, as well as time-of-sleep records. Access to information is one of the essentials for technology to have an empowering effect on patients^13,37,38^. At the same time, we find that older adults continue to only conditionally engage with the SMS giving potential rise for disempowering outcomes when living with SMS.

For the first time since installation of the SMS, participants are able to see their own nighttime data, directly on their smartphones the morning after. This access does not simply add a new feature; it redefines what the system is for several participants. Where the smart mattress had previously functioned as a silent transmitter sending data to relatives and caregivers, it now also transmits data to the person being monitored. Several participants describe this shift in terms that go beyond convenience, framing data access as a form of reclaimed empowerment. P13 mentions, *“now I can see it myself. Before, I did not know if anything was happening. Now I check it in the morning. It is like knowing”* (P13, phase 4). P4 similarly states that seeing their own data changes how they relate to the device, *“I used to feel it was just there for my son. Now I also get something out of it”* (P4, phase 4).

At the same time, participants keep relocating or unplugging devices of the SMS, such as the smoke detection device. Hence, the emerging tension identified is not comprehensively addressed by the mini program rollout. P9 continues to describe managing the smoke alarm as an ongoing practical burden, noting that the device is still positioned away from the kitchen to prevent it from activating during meal preparation (P9, phase 4). The coexistence of this unresolved friction with the new data access feature underlines a structural pattern: material improvements to one component of the system do not automatically resolve behavioral adaptations that stabilize around other devices.

Participants continue the conditional use pattern documented earlier: the mattress is kept disconnected when they feel well, the wristband is not worn. Several offer an explanation grounded in the same self-assessed health rationale encountered previously. P11 states, *“I am fine. I do not need to check every morning. If something is wrong, I will know”* (P11, phase 4).

Among participants the reactance patterns documented in earlier phases stabilize into a background condition. Participants no longer articulate strong objections to the system; they simply keep devices unplugged. Where phase 2 interviews revealed active frustration and explicit reactance, phase 4 interviews suggest that non-use has become routine and therefore less visible as a problem. P10 summarizes this trajectory plainly, *“it is just there. I do not think about it much”* (P10, phase 4). What had initially been a reactive withdrawal from the system has, over time, settled into habitual absence. The system’s protective function is thus absent not because participants actively resist it, but because it has ceased to be a meaningful presence in their daily lives.

Taken together, our findings reveal that the autonomy-safety tension identified across phases takes on new forms with time. While we find our informants claiming autonomy by showing reactance to the system’s constraints, we also find older adults to restructure their daily routines in anticipation of what the system might detect and transmit. Ultimately, we uncover that older adults keep devices in use that they themselves consider unnecessary or not useful, so that the data the devices transmit continues to reassure their relatives. Comparing our findings across participants and phases lifts them out of the chronological sequence in which they first appear and renders them as forms of action, each with its own rationale and its own consequences. Three such forms emerge: reactance, preemptive behavior, and performative compliance. The following section develops them and asks why they produce tensions between autonomy and safety.

## Discussion

While prior work documents unintended consequences of monitoring technologies, it remains unclear how these consequences arise and why they produce tensions between the very values such systems are meant to secure. As set out, what existing accounts leave underspecified is the older adult’s own agency in this process. Our findings address this gap. Rather than passively receiving monitoring, older adults continuously anticipate, interpret, and respond to the arrangement that renders them visible, for instance by relocating devices, adjusting routines, or withdrawing from the system altogether, in order to renegotiate the terms of their own visibility. We refer to this ongoing work as visibility management: the effort through which older adults shape what the system, and through it their relatives and caregivers, can see of them. Explaining this process is what our study adds beyond identifying unintended consequences. We do not offer an inventory of such consequences, but an account of the mechanism that generates them and of why that mechanism produces tensions between the two values of autonomy and safety SMS are supposed to secure simultaneously.

Smart monitoring systems are introduced into community-based elderly care with the intent to enable older adults to live independently and safely in their own homes by providing continuous, unobtrusive protection^12,13^. This intent is operationalized through a set of concrete technological functions. Sensor networks detect falls and monitor movement, smart mattresses record cardiovascular and respiratory activity, among others, and smoke and gas detection devices register environmental hazards, while wristbands continuously track vital signs^7,19^. Crucially, these functions are coupled with alerts and notifications that transmit data and trigger responses across a network of relatives and professional caregivers. It is this coupling, between continuous data collection and third-party access, that constitutes the sociotechnical character of SMS and distinguishes them from passive devices^10,41,51^. While each individual function is designed to serve the older adult’s safety and enable independent living, together they produce a monitoring arrangement in which the older adult’s behavior is continuously rendered visible, interpretable, and actionable by others leading to unintended consequences. We furthermore identify tensions between autonomy, for instance, the ability to make choices and to maintain control over one’s daily life^4^, and safety, that is, the protection from harm, early detection of health risks, and timely intervention^5^. These values are not inherently opposed. Literature on elderly care recognizes tension between autonomy and safety, specifically “for older adults with cognitive impairments, complex health needs, or age-related vulnerabilities”^4, p.1^.

**Fig. 2:**
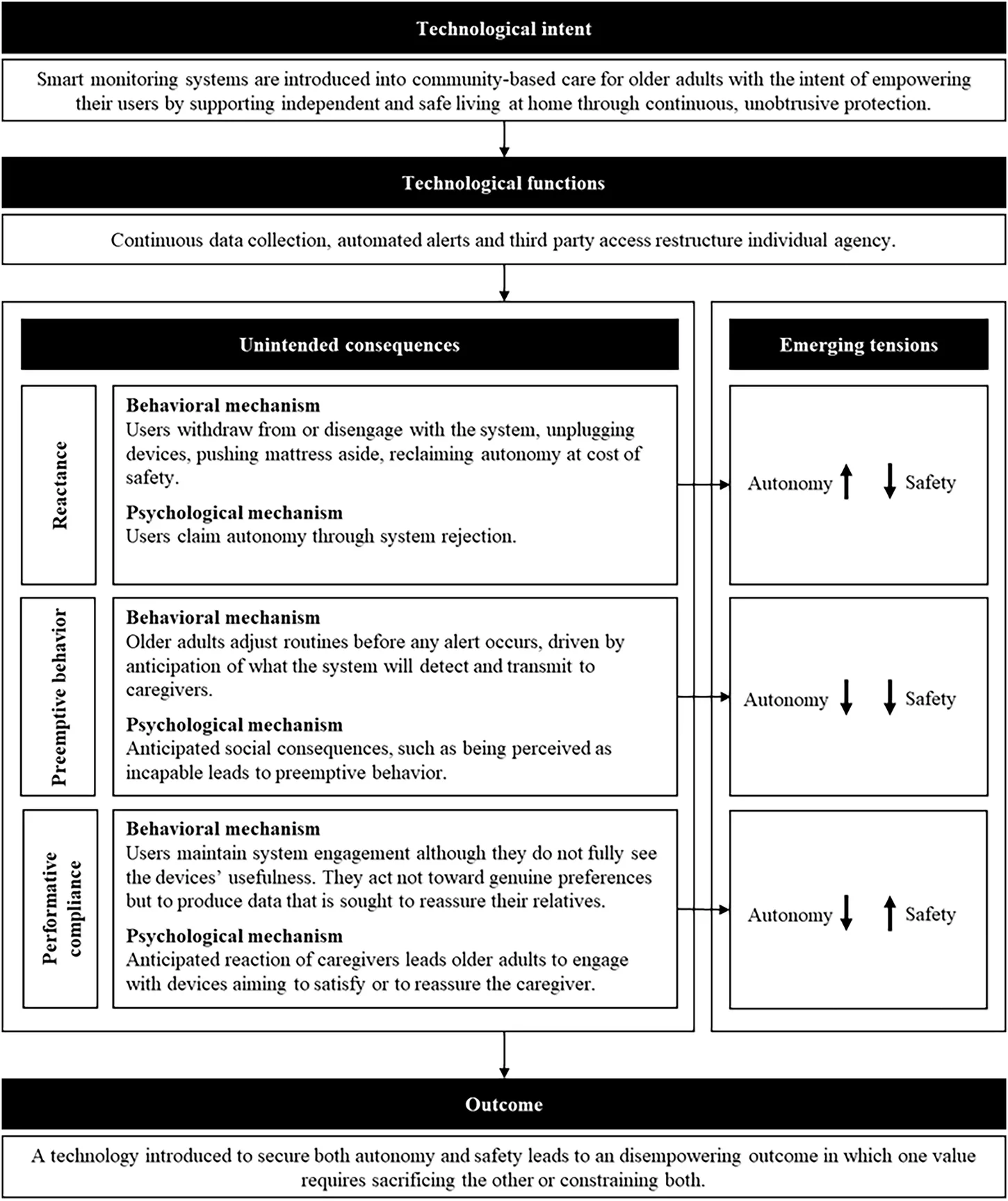
Visibility management: Unintended consequences and value tensions emerging when living with smart monitoring systems.

We theorize reactance, preemptive behavior, and performative compliance as three strategic mechanisms of this single underlying process of visibility management, and we explain how each produces a distinct configuration of the autonomy-safety tension. Over time, older adults claim autonomy by undermining safety (reactance), constrain both (preemptive behavior), or preserve safety by constraining autonomy (performative compliance). We capture these mechanisms in a model (see Fig. 2) and discuss them against existing literature.

First, when living with SMS, *reactance* emerges as the active rejection of the system's constraints. Some informants state that they withdraw from using the system entirely, unplugging devices, pushing the smart mattress aside, or refusing to engage with certain functions. This reactance is not, in most cases, an expression of indifference to safety or as P14 puts it, *“I pushed it [the smart mattress] to the side. I sleep better without it. If something happens, something happens. I don’t want to think about it every night”* (P14, phase 3 A). Research on surveillance in later life has long established that monitoring changes what a home means to those living in it^52,53^ and that resistance to it is meaningful rather than deficient^3^. Our findings build on this work. Where technology adoption research frames such withdrawal as a problem of acceptance or usability, that is, as a failure of the individual to engage adequately with the technology, our findings extend this reading. Older adults deploy distrust deliberately, as a competent response to systems whose behavior they cannot verify^49^. The withdrawal we observe follows that logic: participants do not fail to understand the SMS, they form a judgment about what it does and act on it. Rather than reflecting a deficit in acceptance, reactance to the SMS constitutes an active form of autonomy claiming. Individuals who reject the system are, in effect, trading the safety benefits it offers for independence from its constraints. In doing so, at the cost of the technology’s intended support^12^, the older adults’ safety is undermined in the very act of reclaiming autonomy over their decision-making.

Second, *preemptive behavior* refers to changes users make to their routines in anticipation of health data deviations the SMS detect and transmit to caregivers and relatives. We observe older adults adapting their behavior before any intervention can occur, driven by the desire to avoid triggering the SMS to respond to their behavior. For instance, informants describe cooking on lower heat to prevent smoke detection devices from activating, and adjusting their sleep positions on the smart mattress. Consequently, relocating the smoke and gas detection devices away from the kitchen to prevent false alarms during cooking represents a response that, while eliminating unwanted alerts, simultaneously removes the system’s ability to detect a genuine fire hazard. These are precisely the kind of little arrangements literature identifies as autonomy-enabling in later life: small, locally reasoned modifications through which older adults keep a technology compatible with the way they want to live^50^. In our case, however, the same arrangements carry a second effect that this account does not anticipate, because the object being rearranged is also the one meant to protect the person doing the rearranging. By restructuring everyday behavior to avoid triggering alerts, residents do not merely constrain their autonomy, they also degrade the system’s capacity to ensure safety. Older adults who behave preemptively hence transform the system’s intended function^33^ to the end where neither value is preserved. Autonomy is diminished through self-imposed behavioral restriction, and safety is undermined as the monitoring system is rendered unable to detect the very risks it is designed to identify. Preemptive behavior thus produces precisely the loss of control over one’s own decisions and routines that defines disempowerment^39^ and it does so through the very device introduced to prevent it.

Third, *performative compliance* refers to older adults maintaining engagement with the SMS although they do not fully see the devices’ usefulness for themselves, therefore, producing data that is anticipated to satisfy their caregivers rather than their own preferences or actual wellbeing. They act not toward genuine preferences but to produce data that is sought to reassure their relatives. The performative act of creating an appearance serves to alter how the environment perceives the individual^54^. Residents in this mode continue using devices whose value for themselves they doubt, so that what reaches their relatives conveys that everything is in order. The same orientation can also produce the reverse behavior, where older adults restrict what relatives are able to see, which underlines that what is being managed is not the device but the impression it conveys. Underlying this is the need to demonstrate that one is still capable of living independently and taking care of oneself^8,55^. This demonstration is addressed to a specific audience. The long-term use of a connected device between older adults and their families is organized less around its functional purpose than around the sense of everyday togetherness it sustains: what is transmitted becomes a way of being present to one another^56^. The SMS inverts this arrangement. The data flow is unidirectional and its content is not chosen, so the only way to shape what relatives see is to shape the behavior that produces it. Therefore, performative compliance reveals a distinct form of value tension: unlike preemptive behavior, where both autonomy and safety are compromised, performative compliance preserves safety in a narrow technical sense: no alert is triggered and no intervention occurs but at the direct cost of the older adult’s autonomy. Overall, we propose to not only recognize living with SMS as an empowering factor, but also to acknowledge the process by which a technology intended to empower can, due to its specific technical functions, lead to unintended consequences and resulting value tensions. These tensions can result in an overall disempowering outcome that contradicts the original technological intention.

The process we theorize describes settings in which monitoring is institutionally deployed rather than individually chosen; in which alerts are routed to third parties, whether relatives or professional caregivers, rather than to the monitored person; in which devices are physically present in the home and their operation is perceptible to the person living with them; and in which the older adult does not perceive an acute health risk that makes continuous protection self-evidently desirable. Where these conditions do not hold, we would expect the mechanisms we identify to weaken or to take a different form, and our own material offers two indications of this: where data are returned to the monitored person, as in phase 4, the interpretation of the system shifts while adaptations that have already stabilized persist; and where an acute health risk is present, as in the case of P9, safety is experienced as a precondition of independence rather than as a constraint on it. Our empirical basis is one housing complex, one device configuration, and one year of use within a single national care context, which further bounds the claims we make.

This study contributes to health IS literature, the sociotechnical understanding of health technology^14,33^, and literature on value tensions in care contexts^34,35^ in two ways. First, addressing the lack of longitudinal research on SMS use, we provide a longitudinal empirical account of living with SMS that captures the behavioral and psychological mechanisms through which unintended consequences unfold over time. Prior health IS research has primarily focused on enhancing technological performance to improve care delivery^17–19^, implicitly treating monitoring as a passive process in which data flows unobtrusively to caregivers and alerts trigger responses^5,20,21^.Our findings show that being continuously monitored is instead an anticipatorily active experience: older adults are aware of being observed and continuously adjust their behavior in response to how they believe the system and its responders interpret them. Crucially, the QLR design^24,26,27^ reveals that these responses are not stable reactions but a trajectory that cross-sectional accounts of anticipated and unanticipated consequences^22^ cannot capture. Initial acceptance grounded in institutional trust gives way to reactance triggered by discomfort and false alarms, then to conditional use and device relocation, before non-use finally settles into habitual absence in which the system’s protective function fades without articulated resistance. Following individuals from installation onward thus allows us to observe how early frictions stabilize into enduring behavioral patterns rather than reconstructing them retrospectively^32^.

Second, we offer a conceptual contribution by identifying anticipation and behavioral adjustment as the mechanisms that produce autonomy-safety value tensions, thereby challenging the prevailing assumption that SMS jointly optimize autonomy and safety as an inherently empowering outcome^4,12,13^. While literature on elderly care recognizes tensions between autonomy and safety in principle^4,47^, it leaves unspecified how such tensions arise in everyday use. We show that they are enacted through the older adult’s own visibility management: anticipating what the system might detect and transmit, users respond through reactance, preemptive behavior, or performative compliance, each producing a distinct configuration of the tension. Reactance claims autonomy by undermining safety, performative compliance preserves safety in a narrow technical sense at the cost of autonomy, and preemptive behavior constrains both values simultaneously. This conceptualization extends value tensions literature^34,44,46^ by demonstrating that these tensions require no technological malfunction: they emerge from the system’s intended functioning, that is, the coupling of continuous data collection with alerts and third-party data accessParticipants and recorded conversation. It also reframes responses that prior work treats as acceptance or usability problems^3^ as purposeful strategies through which older adults renegotiate the terms of their own visibility^48–50^.

Our findings carry practical implications for designing monitoring systems that support communication, agency, and negotiated control for older adult users. The identified unintended consequences reveal that the mere installation of monitoring technology is insufficient and may even be counterproductive: older adults actively shape what the system reports out of anticipation of how they are seen.

First, our findings point to designing SMS that support communication rather than replace it. Alerts routed exclusively to relatives disrupt family relationships and provoke disengagement, as participants prefer to be informed of deviations themselves rather than having their children alarmed on their behalf. This suggests configurable alert routing that notifies the older adult first where possible and escalates to caregivers only when no response occurs. For informal caregivers, this implies treating alerts and behavioral data not as objective indicators of safety or incapacity but as a starting point for conversation about what the data means and what support is actually needed.

Second, our findings highlight the importance of strengthening older adults’ agency over their own monitoring. The phase 4 rollout of smartphone access to nighttime data specifically illustrates this potential: once participants have access to their own data, the mattress shifts from a device serving others to one older adults understand as serving their own safety. This resonates with the argument that human-data interaction with older adults should be designed around what people want to know about themselves rather than around what a system is able to measure about them^57^. Our phase 4 material points to the same design move and adds a temporal qualification: returning data to the person being monitored changes how the system is understood, but it does not undo behavioral adaptations that have already stabilized around other devices. Deployment therefore calls for accompanying structures that give older adults access to information about the system and about themselves, alongside dignified support in which questions are clarified and functions are presented transparently.

Third, SMS benefit from allowing negotiated control. Participants in our study relocate, unplug, and conditionally use devices unilaterally and often invisibly, undermining the system’s protective function without anyone noticing. Rather than treating such adaptations as misuse, providing legitimate channels for negotiation, that is, adjusting sensor sensitivity, repositioning devices, or deactivating individual components in dialogue with the older adult, makes these choices explicit and revisable. In this way, the trade-off between autonomy and safety remains a matter of ongoing, shared decision-making.

Our study offers early evidence on a critical knowledge gap, that is, unintended consequences when living with SMS. However, its claims are bounded by the scope conditions stated above. Beyond these, four limitations apply to what our design could observe. The study takes place in a single community-based housing setting in one national context, which, while enabling analytical depth, constrains the direct transferability of findings to other care arrangements or technology configurations where autonomy and safety may be negotiated differently. Additionally, interview-based methods may exclude those experiencing cognitive, physical, or access-related constraints and some residents in our setting could not be interviewed for these reasons. This circumstance introduces a systematic gap in our data: precisely those individuals most vulnerable to disempowering dynamics we describe may be least reachable through interview-based methods. Furthermore, our analysis draws only on what we could observe and on what participants articulated. However, the SMS continuously generates sensor readings and event logs. These data are held by the deploying institutions, were not covered by the consent obtained from participants, and were not available to the research team. They therefore lie outside our analyzed corpus. As a consequence, we can account for how participants describe and enact their responses to being monitored, but we cannot corroborate these accounts against the system’s own record of activity, nor determine whether behaviors such as alert preemption register as detectable shifts in sensor patterns. Last, we cannot yet establish how both values, autonomy and safety can be jointly secured. This is the central open question that emerged from our findings. Future research should therefore investigate what conditions in the technology’s design, in the relational and institutional context of deployment, and in the characteristics of individual users enable the joint advancement of both values rather than the trade-off between them or the restriction of both. Therefore, future work employing mixed-methods designs combining observational data, system logs, and longitudinal interviews across diverse care settings could expand our knowledge on value tensions when living with SMS.

## Methods

**Fig. 3:**
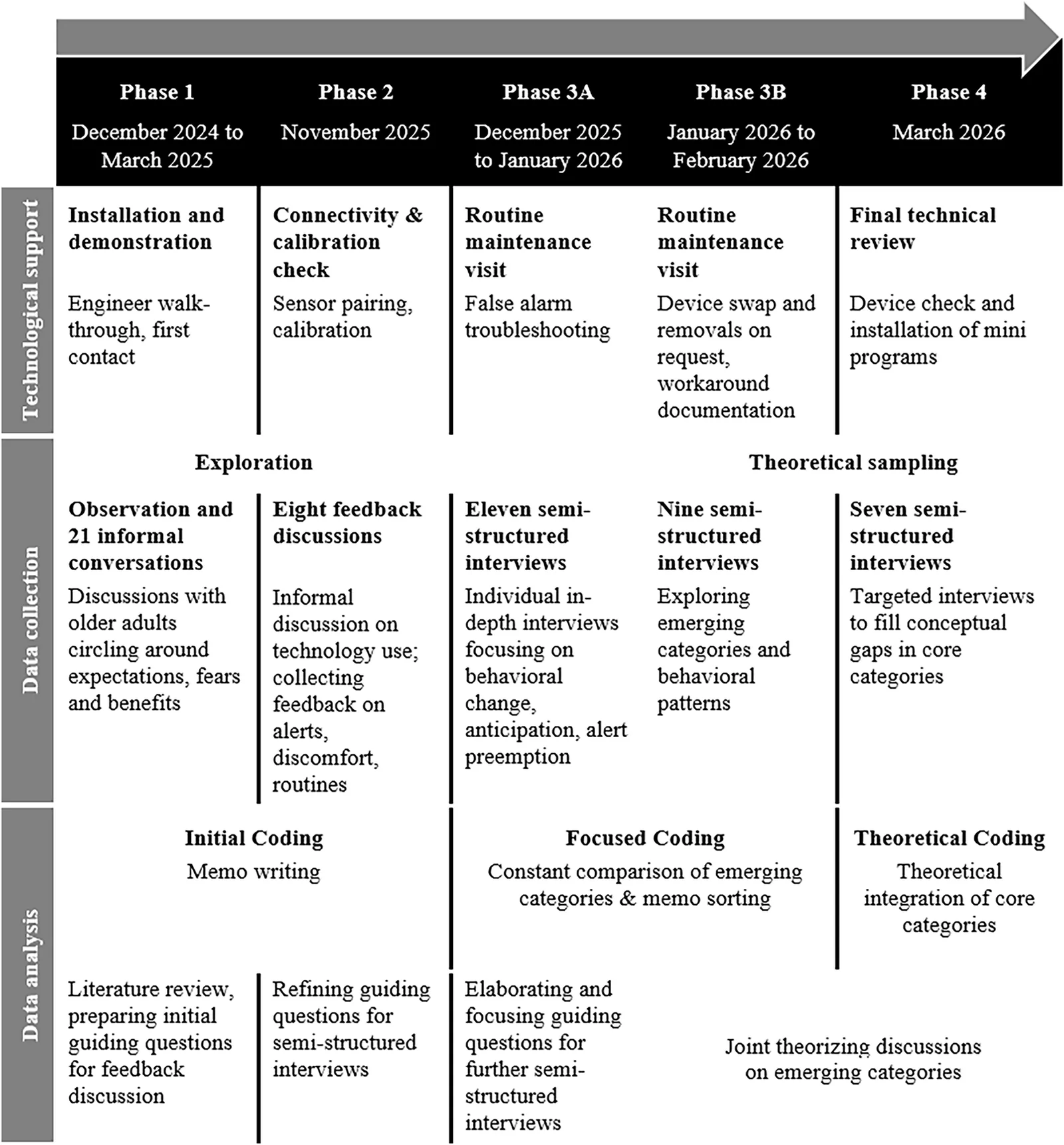
Qualitative longitudinal research design

To approach our research question, we followed an inductive QLR design^24,26,27^. This approach (see Fig. 3) was particularly suitable for two reasons. First, behavioral and psychological consequences of living with SMS gradually unfold as individuals experience continuous monitoring in their homes. Capturing this emergence called for engagement that extends over time rather than a single point of measurement^27^. Second, QLR allowed us to capture how routines changed as they happen, rather than reconstructing them retrospectively^32^. We analyzed our data using an iterative coding approach, in which categories were built inductively from the data and refined through constant comparison across participants and time points, with literature serving as a sensitizing lens^25,29^.

### Community-based housing for older adults in China

We conducted our study in a community-based housing complex for older adults comprising 522 households. Residents maintained their own private rooms and lived independently, with regular support available from on-site healthcare workers as well as relatives, including children and grandchildren, some of whom also resided in the near community. The housing complex accommodated a mixed population of older adults, some of whom were military veterans assigned to the complex through a regional administration center. The smart monitoring initiative was developed and managed by two Chinese universities and the community management center. At the community level, a street-level municipal office facilitated the project implementation and maintained operational knowledge of the system within the neighborhood.

**Fig. 4:**
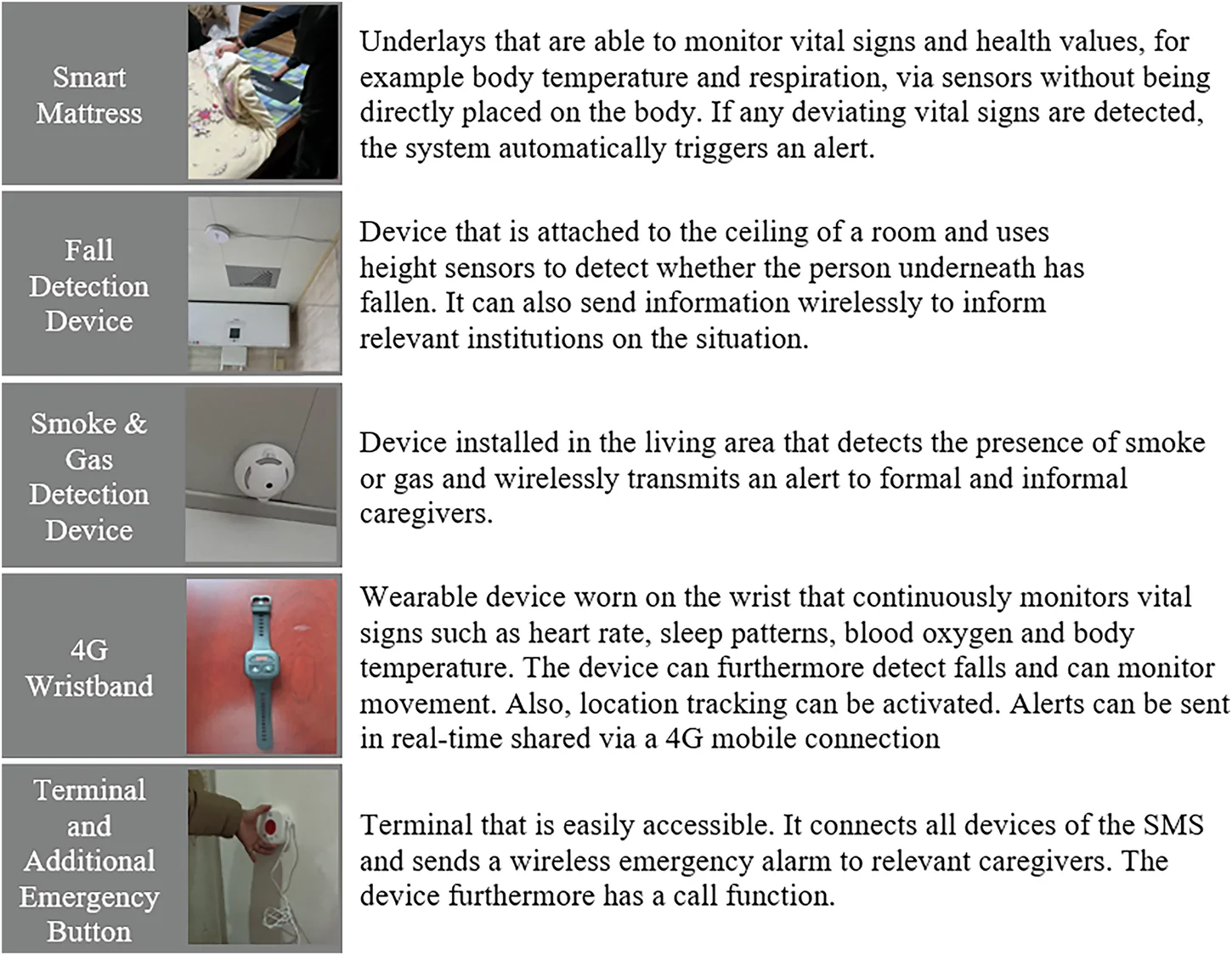
Installed devices of the smart monitoring system under investigation.

During the study period, five types of smart monitoring devices were installed across 44 apartments (see Fig. 4). These devices ranged from smart mattresses continuously recording cardiovascular signs and respiratory rates with automated deviation-triggered alerts transmitted via mobile connection to 4G wearable wristbands tracking heart rate and other vital signs. Also, infrared fall detection sensors were deployed across multiple rooms to detect movement patterns, and home gateway terminals were intended to enable residents to call for assistance at any time. Lastly, smart smoke detectors with remote alert functionality were installed, as well. While most residents accepted all available devices to be installed, some requested additional fall detection sensors, and a small number of residents decline smoke and gas detection devices.

### Data collection: longitudinal qualitative study

Over a 15-month period from December 2024 to March 2026, we followed a four-phase design iteratively combining interview data collection and analysis. This inductive logic in which early observations and semi-structured interviews informed the direction of subsequent data collection was central to our approach and allowed emerging patterns to be pursued as they surfaced. Overall, we recruited 21 older adults between 75 and 87 years old, all of whom we accompanied during installation in phase 1 (see Table 1). Nineteen took part in at least one recorded conversation in the subsequent phases, twelve of them more than once across up to four phases, while two withdraw after phase 1. Not all participants were therefore interviewed at every data collection point. As some participants were temporarily unavailable due to health episodes, later interview rounds were guided by emerging themes^58^. Therefore, certain participants were prioritized because their experiences were particularly relevant to developing our analytical categories^29^. The research project received ethical approval for the collection of primary data, and written informed consent was obtained from all participants.

**Table 1:** Participants and recorded conversation durations by phase.

#	Age in years	Phase 1 December 2024 to March 2025	Phase 2 November 2025	Phase 3 A December 2025 to January 2026	Phase 3B January 2026 to February 2026	Phase 4 March 2026
		Installation and demonstration	Connectivity & calibration check	Routine maintenance visit	Routine maintenance visit	Final technical review
P1	79	174 m 16 s total duration of informal conversations^a^	13 m 14 s	-	61 m 43 s	-
P2	85		04 m 51 s	-	-	-
P3	83		03 m 11 s	-	-	-
P4	79		05 m 34 s	15 m 41 s	34 m 50 s	10 m 58 s
P5	83		07 m 11 s	-	-	-
P6	83		05 m 53 s	27 m 05 s	-	-
P7	85		12 m 18 s	-	40 m 28 s	-
P8	81		03 m 42 s	-	24 m 26 s	-
P9	87		-	14 m 26 s	-	06 m 06 s
P10	78		-	14 m 26 s	-	07 m 23 s
P11	83		-	13 m 36 s	-	05 m 22 s
P12	76		-	26 m 51 s	-	09 m 57 s
P13	80		-	13 m 54 s	51 m 12 s	19 m 57 s
P14	85		-	15 m 10 s	40 m 36 s	-
P15	83		-	42 m 32 s	-	-
P16	75		-	14 m 45 s	-	-
P17	83		-	15 m 52 s	61 m 08 s	16 m 21 s
P18	81		-	-	39 m 46 s	-
P19	78		-	-	24 m 59 s	-
P20	80		-	-	-	-
P21	78		-	-	-	-

^a^Phase 1 is reported as an aggregate because conversations during installation were fluid and overlapping and cannot be segmented into individually timed interviews.

In phase 1 (December 2024 to March 2025), we shadowed technicians as they installed the respective monitoring devices in individual apartments. During installation, we observed how residents reacted to the technology being set up. This phase specifically served two purposes: it provided a direct first impression of how residents encountered the technology, and it helped us understand the technical functions of each device. For privacy reasons, we recorded observations through field notes, photographs, and audio memos^30^. Furthermore, informal conversations during the installation provided us with insights into the informants’ expectations and concerns.

Following installation, we engaged with literature on aging scholarship and monitoring technology to develop an initial understanding of our observations^29^. Next, we formulated guiding questions that served to structure a first round of feedback discussions with eight participants in phase 2^59^. Within these discussions, we captured the older adults’ first extended experience of living with their SMS. By this point, residents integrated or chose not to integrate the devices into their daily routines, making this a critical moment to surface early behavioral and psychological responses to the technology. Afterwards, we refined our interview guidelines (see supplementary material), pursuing emerging themes.

Hence, in phase 3 A (December 2025 to January 2026) and 3B (January to February 2026), we returned to the housing complex to conduct semi-structured individual interviews. Participants then lived with the SMS in their daily routines for almost one year, allowing us to explore how initial perceptions had evolved, whether and how behavior had changed, and what tensions had emerged over time^32^. The subdivision of this phase was primarily based on the availability of the informants. While some were unavailable in phase 3 A, we were able to speak with them in phase 3B. Additionally, in phase 3B, we had the opportunity to discuss more in-depth questions with informants we spoke to in phase 3A^29^. Lastly, in phase 4, we conducted a final round of semi-structured interviews with a theoretically sampled subset of participants to specifically capture how participants navigated the balance between autonomy and safety over time.

In phase 1, we retrieved field notes, photographs and audio memos recorded while shadowing installations, together with 21 informal conversations (2 h 54 min in total). These were treated as ethnographically embedded material and used for initial coding and memo writing. Across phases 2 to 4, our data collection yielded 35 recorded conversations: 8 short feedback discussions in phase 2 (mean 7 min), 11 interviews in phase 3 A (mean 20 min), 9 in-depth interviews in phase 3B (mean 42 min), and 7 follow-up interviews in phase 4 (mean 11 min). Conversation length thus varied systematically with the purpose of each phase, from brief early reactions to extended accounts once participants had lived with the system for close to a year. Together with the 2 h 54 min of informal conversation recorded during installation, the corpus comprised 15 h of recorded material and 378 pages of transcript. Across all phases, conversations with our informants were conducted in Chinese language by native-speaking members of the research team, transcribed verbatim, and translated into English for analysis. Translation was also performed by bilingual team members who had themselves conducted the conversations, so that contextual knowledge of the setting and the interaction informed the translation procedure accordingly^60,61^. Joint theorizing sessions and the accompanied memo-writing were therefore also conducted in English language.

Sample size in a design of this kind is governed by information power rather than by participant numbers^62^: the more narrowly bounded the phenomenon and the denser the engagement with each participant, the fewer participants are required. Our study was narrowly bounded, covering one housing complex, one device configuration, and one year of use, and our engagement was dense and situated. Older adults living with monitoring technology are moreover a hard-to-reach population, in which recruitment and retention are constrained by cognitive impairment, physical frailty, and willingness to participate across an extended period. We therefore pursued analytical rather than statistical generalization^63^, moving inductively from situated observations toward patterns and categories that account for the phenomenon under study^32^.

### Data analysis

Our analysis drew on the full corpus described above: field notes, photographs, and audio memos from phase 1 together with the informal conversations recorded during installation, 35 transcribed conversations from phases 2 to 4, and coding memos and records of the joint theorizing sessions in which emerging categories were challenged across the research team. The monitoring system also generated sensor and log data. These were held by the deploying institutions, were not covered by the consent obtained from participants, and were not available to the research team; they therefore fell outside the analyzed corpus. All collected data was iteratively analyzed and coded, constantly comparing emerging themes and categories^29^. Therefore, analysis proceeded in three steps.

First, we conducted initial coding, working through our ethnographic material, that is, field notes, photographs, and audio memos, to capture and identify initial reactions to the installation of SMS devices, questions the participants ask, and perceptions in participants’ expressions. Given the observational character of this material, we coded incident by incident, and formulated codes as gerunds in order to keep participants’ actions in view^29^. This granular engagement with our data kept our initial understanding close to participants’ expressed experiences^28^.

Second, we pursued focused coding, in which we grouped initial codes into higher-order categories based on their conceptual relationships. At this stage we worked primarily with our conversational corpus of audio-recorded and verbatim transcribed conversations, namely feedback discussions (Phase 2) and semi-structured individual interviews (Phases 3 A, 3B, and 4). This material carried the analysis forward because it spanned the full observation period and thus permitted comparison across time. Focused coding involved constant comparison^28^: systematically comparing incidents within and across participants and across time points, we identified recurring patterns and variations^24,26^. Codes such as *cooking on lower heat*, *pushing away the mattress*, and *avoiding certain rooms* were grouped under the category of *behavioral adjustment*, which in turn was related to broader categories such as *alert preemption* and *performative compliance*.

Third, we engaged in theoretical coding connecting emerging categories with concepts from literature^29^ on sociotechnical understanding^7,14^, and value tensions^34,35,45^ to interpret the relationships between categories and to develop our core arguments^30,31^. Importantly, theory was used as a sensitizing lens^25^. Moreover, it guided our attention toward dynamics while remaining open to patterns that did not fit pre-existing concepts^63^. Throughout the analysis, we held regular joint theorizing sessions in which members of the research team compared and discussed their interpretations to challenge emerging categories. Records of the joint theorizing sessions in which categories were challenged across the team further informed our analysis^29^.

## Supplementary information

Supplementary information

## Data Availability

The data collected and analyzed in the course of the study are not publicly available as they fall under data protection constraints. They include sensitive qualitative interview data. Anonymized and aggregated data supporting the findings of the study may be made available upon reasonable request.
