# Supplementary material for: Renegotiating autonomy and safety: disentangling tensions emerging for older adults living with smart monitoring systems
Source: Npj Health Syst. 2026 Oct 8;3:96. doi: 10.1038/s44401-026-00154-4 (PMC13649674; doi:10.1038/s44401-026-00154-4)
Supplement: Supplementary file 1 [file 44401_2026_154_MOESM1_ESM.pdf]

Supplementary material

Exemplary Interview Questions from Phase 2 and Phase 4

Phase 2

1. What kind of monitoring devices do you use?
    a. Why do you use the SMS?
    b. What are the specific functions of each of the device that you use?
2. Who exactly gets access to your data that is stored and forwarded via the technology?
    a. Who decided that this specific device was implemented in your apartment?
    b. How were you involved in the decision-making process?
    c. How do you feel about the decision of the technology being implemented in your apartment?

Phase 4

3. It has been over a year now since the monitoring system was installed in your apartment. When you think about daily life today compared to when it was first put in, what, if anything, feels different?
    a. How does it feel different?
    b. What exactly feels different?
    c. Why does it feel different?
4. Is there anything you do differently, now that the smart monitoring system is installed?
5. When you go about your day (sleeping, cooking, moving around your apartment), do you find yourself thinking about the monitoring system? Please walk me through a recent situation where it came to mind.
    a. Who were you thinking about in that moment (the device itself, a caregiver, a family member)?
    b. Why did you think about them?
